# Normalization of the levels of inflammatory molecules in *Mycobacterium smegmatis*-infected U937 cells by fibrate pretreatment

**DOI:** 10.1186/0717-6287-47-42

**Published:** 2014-09-15

**Authors:** Sung-Jo Kim, Minho Hong, Ki Duk Song, Hak-Kyo Lee, Sungweon Ryoo, Tae-Hwe Heo

**Affiliations:** Department of Biotechnology, Hoseo University, 165, Baebang, Asan, Chungnam, 336-795 Republic of Korea; The Animal Genomics and Breeding Center, Han-Kyong National University, Anseong, 456-749 Republic of Korea; Korean Institute of Tuberculosis, Mansu-ri 482, Gangoe-myeon, Cheongwon-gun, Chungcheongbuk-do, 363-954 Republic of Korea; Laboratory of Immunology, Integrated Research Institute of Pharmaceutical Sciences, College of Pharmacy, The Catholic University of Korea, Bucheon, 420-743 Republic of Korea; NP512, Hall of Cardinal Jin-Suk Cheong, The Catholic University of Korea, 43 Jibong-ro, Wonmi-gu, Bucheon-si, Gyeonggi-do, 420-743 Republic of Korea

**Keywords:** Fibrate, MyD88, TNF-α, Tuberculosis

## Abstract

**Background:**

Tuberculosis (TB) is a respiratory tract disease caused by *Mycobacterium tuberculosis* infection. *M. tuberculosis* exploits immune privilege to grow and divide in pleural macrophages. Fibrates are associated with the immune response and control lipid metabolism through glycolysis with β-oxidation of fatty acids.

**Results:**

In this study, we investigated the effect of fibrate pretreatment on the immune response during *M. smegmatis* infection in U937 cells, a human leukemic monocyte lymphoma cell line. The protein expression of tumor necrosis factor α (TNF-α), an inflammatory marker, and myeloid differentiation primary response gene 88 (MyD88), a toll like receptor adaptor molecule, in the infected group increased at 1 and 6 h after *M. smegmatis* infection of U937 cells. Acetyl coenzyme A acetyl transferase-1 (ACAT-1), peroxisome proliferator-activated receptor-α (PPAR-α), TNF-α, and MyD88 decreased in U937 cells treated with fibrates at 12 and 24 h after treatment. More than a 24 h pretreatment with fibrate resulted in similar expression levels of ACAT-1 and PPAR-α between infected vehicle control and infected groups which were pretreated with fibrate for 24 h. However, upon exposure to *M. smegmatis,* the cellular expression of the TNF-α and MyD88 in the infected groups pretreated with fibrate for 24 h decreased significantly compared to that in the infected vehicle group.

**Conclusion:**

These results suggest that fibrate pretreatment normalized the levels of inflammatory molecules in *Mycobacterium smegmatis*-infected U937 cells. Further studies are needed to confirm the findings on pathophysiology and immune defense mechanism of U937 by fibrates during *M. tuberculosis* infection.

## Background

*Mycobacterium tuberculosis* causes tuberculosis (TB) infection, a disease which kills two million people every year [1–3]. Phagocytosis of *M. tuberculosis* by alveolar macrophages results in the accumulation of oxidized low-density lipoproteins which provides immune privilege to the pathogen. *M. tuberculosis* exists inside a granuloma, a hallmark of TB. However, the underlying mechanism of TB pathogenesis is not fully understood [4–6]. Because *M. tuberculosis* is highly infectious and must be handled in a biosafety level-3 or above facility, research on the mechanism of TB infection has generally been performed using alternative mycobacterium species such as *M. smegmatis, M. bovis, Bacillus calmette-guerin* (BCG) or *M. marinum* [7–10]. Detection of mannose-6-phosphate isomerase class I (ManA) and methionine amino peptidase (MetAP) using polymerase chain reaction (PCR) has been performed to diagnose mycobacteria infection in macrophages [4, 11–13]. Furthermore, ManA [14] and MetAP [12] from *M. tuberculosis* have been regarded as promising antituberculosis targets. Fibrates affect lipid and lipoprotein metabolism through activating transcription factors including peroxisome proliferator-activated receptor-α (PPAR-α) [15]. About 80 fibrate substances are synthesized from dehydrocholic acid, phenylethyl acetic acid, and other acetic acids and studies have shown the effects of hypocholesterolemia in both humans and rats [16–19]. Several fibrates have been developed, including bezafibrate, fenofibrate, and gemfibrozil. Fenofibrate and gemfibrozil have been used in North America and bezafibrate and ciprofibrate in Europe. The direct effect of fibrates on TB infection have not been studied well. Among fibrates, it was reported that gemfibrozil, a commonly prescribed hypolipidemic drug, blocked TB growth by inhibiting enoyl reductase [20] and bezafibrate differentiates human myeloid leukemia cells [21].

PPAR-α belongs to the large PPAR nuclear receptor superfamily and forms a heterodimer with the 9-cis-retinoic acid receptor (RXR) to bind to the peroxisome proliferator response element complex, which is a transcriptional regulatory element controlling lipid and carbohydrate metabolism with hypolipidemic effects [22–25]. In addition, PPAR-α is involved in endothelial dysfunction, myocardial ischemic injury, and immune-inflammatory responses in cells [26]. Cholesterol levels of host cells have an effect on TB infection, as a high level of cholesterol in the diet contributes to increase in TB infection rate [16, 18, 19]. In addition, TB is known to aggress upon host cells via cholesterol-rich membrane microdomains [2, 3, 27]. Despite the possible mutual link between TB and PPAR-α via lipid metabolism, the direct involvement of PPAR-α in TB pathogenesis is still obscure.

In the case of bacterial infection including TB, several immune response factors, such as nuclear factor kappa beta (NF-κB) and tumor necrosis factor-α (TNF-α) in human monocytes, play important roles in innate immunity and function, leading to migration of NF-κB into the nucleus by immune signal transduction via TLR2 [28, 29]. Especially, TNF-α is involved at multiple steps in immune response to *M. tuberculosis* [30]. In addition, myeloid differentiation primary response gene 88 (MyD88) is important for triggering macrophage effector mechanisms against *M tuberculosis* [31]. PPAR-α activator, gemfibrozil, has shown the inhibitory effect of TNF-α partly by antagonizing NF-κB in neonatal rat cardiac myocytes [32].

Acetyl coenzyme A acetyl transferase-1 (ACAT-1) is a mitochondrial enzyme that participates in the degradation and formation of ketone bodies, and synthesis of acetoacetyl-CoA using two acetyl-CoAs [5, 6, 33, 34]. Acetoacetyl-CoA is converted to cholesterol in the early cholesterol biosynthetic pathway [8–10, 35, 36]. Despite the possible mutual link between TB and ACAT-1 via lipid metabolism, the direct involvement of ACAT-1 in TB pathogenesis is still obscure.

In this study, we intended to investigate the impact of fibrates treatment on *M. smegmatis* infected cells, and observed that pretreatment of differentiated U937 cells with fibrate affects the expression of PPAR-α, which is a target receptor of fibrates, myeloid differentiation primary response gene 88 (MyD88), which participates in the immune response, and acetyl coenzyme A acetyl transferase-1 (ACAT-1), which participates in TNF-α activation and early cholesterol pathway, consequently alleviated infection by *M. smegmatis* in macrophages.

## Results

### Identification of M. smegmatis infection

**Figure 1:**
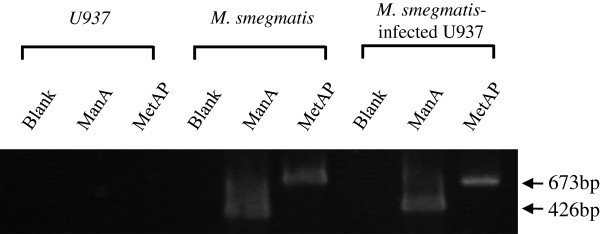
***Mycobacterium smegmatis*** **infection analysis performed by polymerase chain reaction.** mannose-6-phosphate isomerase class I (ManA) and methionine amino peptidase (MetAP) bands were detected using specific primers with bacterial genomic DNA or that from *M. smegmatis*-infected U937 cells. B.

The U937 cells differentiated by PMA were exposed to *M. smegmatis* to identify the infection. After lysis of differentiated U937 cells, the collected genomic DNA was used with a specific primer designed for mannose-6-phosphate isomerase class I (ManA) and methionine amino peptidase (MetAP), which are both specifically expressed in *M. smegmatis*. ManA and MetAP bands were observed, and, thus, *M. smegmatis* infection in the U937 cells was confirmed (Figure 1).

### Differential expression of ACAT-1, PPARα, TNF-α, and MyD88 in U937cells infected with M. smegmatis

**Figure 2:**
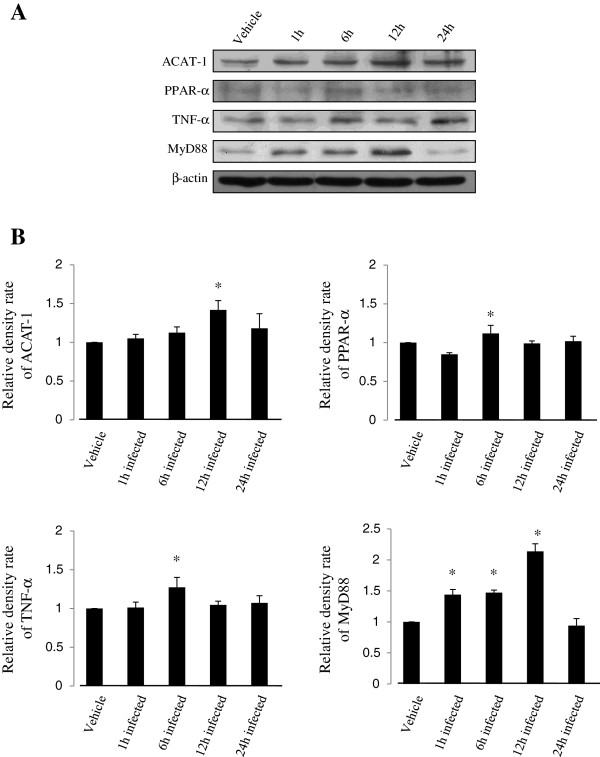
**Fibrate pretreatment affects acetyl coenzyme A acetyl transferase-1 (ACAT-1), peroxisome proliferator-activated receptor-α (PPAR-α), tumor necrosis factor-α (TNF-α), and myeloid differentiation primary response gene 88 (MyD88) expression patterns in U937 cells. (A)** The expression patterns of various factors at 12 h or 24 h are shown after treatment with fibrates. The expression patterns associated with all factors decreased after 12 h and 24 h compared with those in the untreated control. β-actin was used as the loading control. **(B)** Each protein density of fibrate-treated cells were normalized to that of untreated vehicle cells. Each result represents the mean ± SD of three experiments. Significance was calculated as *p value < 0.05 compared to the control by Student’s unpaired *t*-test. Beza, bezafibrate; Feno, fenofibrate; Gem, gemfibrozil.

Western blotting was performed to identify the effect of *M. smegmatis* infection in U937 cells by examining the expression of ACAT-1, PPARα, TNF-α, and MyD88 (Figure 2). The expression level of ACAT-1 in the infected group was significantly different at 12 h after infection compared to that of the non-infected vehicle control (ACAT-1, 1 h infection; 1.05 ± 0.05, *p* > 0.01, 6 h infection; 1.12 ± 0.07, *p* > 0.01, 12 h infection; 1.42 ± 0.05 p < 0.05, 24 h infection; 1.18 ± 0.19, *p* > 0.05), whereas PPAR-α expression decreased slightly at 1 h after *M. smegmatis* infection (PPAR-α, 1 h infection; 0.85 ± 0.02, *p* < 0.05, 6 h infection; 1.12 ± 0.10, *p* > 0.05, 12 h infection; 0.99 ± 0.03, *p* > 0.05, 24 h infection; 1.02 ± 0.06, *p* > 0.05) and increased at 6 h after *M. smegmatis* infection. TNF-α expression increased by about 30% at 6 h and then decreased to normal expression levels at 24 h. (TNF-α, 1 h infection; 1.01 ± 0.07, *p* > 0.05, 6 h infection; 1.27 ± 0.13, *p* < 0.05, 12 h infection; 1.05 ± 0.05, *p* > 0.05, 24 h infection; 1.07 ± 0.09 p > 0.05) MyD88 expression increased significantly at 1, 6, and 12 h after *M. smegmatis* infection. (MyD88, 1 h infection; 1.44 ± 0.08, *p* > 0.05, 6 h infection; 1.47 ± 0.04, *p* < 0.01, 12 h infection; 2.14 ± 0.12, *p* < 0.01, 24 h infection; 1.18 ± 0.19, *p* > 0.05).

### Expression of ACAT-1, PPAR-α, TNF-α, and MyD88 after U937 cell pretreatment with fibrates

**Figure 3:**
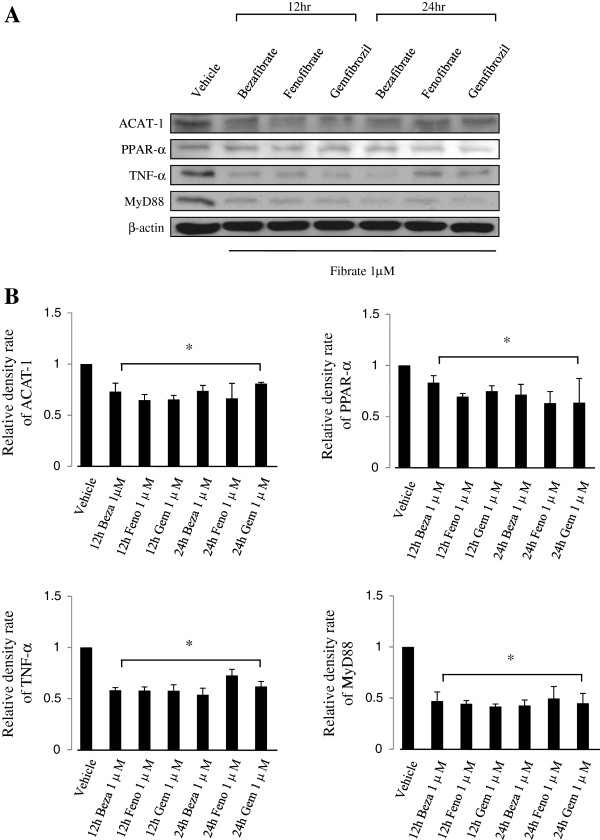
**Fibrate pretreatment affects acetyl coenzyme A acetyl transferase-1 (ACAT-1), peroxisome proliferator-activated receptor-α (PPAR-α), tumor necrosis factor-α (TNF-α), and myeloid differentiation primary response gene 88 (MyD88) expression patterns in U937 cells. (A)** The expression patterns of various factors at 12 h or 24 h are shown after treatment with fibrates. The expression patterns associated with all factors decreased after 12 h and 24 h compared with those in the untreated control. β-actin was used as the loading control. **(B)** Each protein density of fibrate-treated cells were normalized to that of untreated vehicle cells. Each result represents the mean ± SD of three experiments. Significance was calculated as *p value < 0.05 compared to the control by Student’s unpaired *t*-test. Beza, bezafibrate; Feno, fenofibrate; Gem, gemfibrozil.

Bezafibrate, fenofibrate, and gemfibrozil were selected among the commonly available fibrates to study their effects on differentiated U937 cells. Western blotting was used to identify ACAT-1, which participates in early cholesterol synthesis, PPAR-α, which is a fibrate receptor, TNF-α, which is a inflammatory cytokine, and MyD88, which is a promoter of signal transduction for TB and binds to the Toll like receptor 2 (TLR2) membrane protein (Figure 3). ACAT-1, PPAR-α, and TNF-α expression levels decreased about 15–35% compared with that in the vehicle control following treatment with bezafibrate, fenofibrate, or gemfibrozil. MyD88 in U937 cells treated with fibrates decreased by about 50% compared with that in the control group, and the decrease was generally maintained for 24 h or more.

### The effect of fibrate pretreatment on ACAT-1, PPARα, TNF-α, and MyD88 protein expression in U937 cells infected by M. smegmatis

ACAT-1 protein expression in the infected group increased slightly at 6 h after infection compared to that in the non-infected vehicle control, but the difference was not significant. PPAR-α protein expression was also not significantly different between the infected and vehicle control groups and the fibrate pretreated group.

However, TNF-α protein expression decreased about 50% in the bezafibrate, 55% in the fenofibrate, and 25% in the gemfibrozil treated groups compared with that in the non-infected vehicle control group at 1 h after infection. At 6 h, the TNF-α had decreased about 35% in the bezafibrate, 40% in the fenofibrate, and 25% in the gemfibrozil treated cells (1 h infection: 24 h bezafibrate treatment; 0.49 ± 0.13, *p* < 0.01, 24 h fenofibrate treatment; 0.44 ± 0.12, *p* < 0.01, 24 h gemfibrozil treatment; 0.75 ± 0.13, *p* < 0.05, 6 h infection: infected control; 1.26 ± 0.12, *p* < 0.05, 24 h bezafibrate treatment; 0.65 ± 0.17, *p* < 0.05, 24 h fenofibrate treatment; 0.58 ± 0.15, *p* < 0.01, 24 h gemfibrozil treatment; 0.74 ± 0.13, *p* < 0.05).

**Figure 4:**
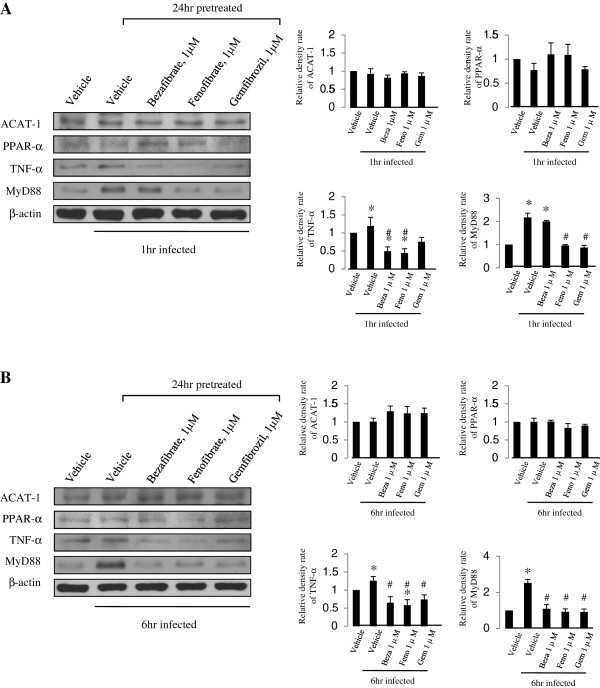
**Tumor necrosis factor-α (TNF-α) and myeloid differentiation primary response gene 88 (MyD88) protein expression levels were regulated by pretreatment with fibrates (1 μM concentration, 24 h) in** ***M smegmatis*** **-infected U937 cells.** Results at 1 h after infection **(A)** and 6 h after infection **(B)**. β-actin was used as the loading control. Each protein density of fibrate-treated or untreated (vehicle) *M smegmatis*-infected cells were normalized to that of uninfected vehicle cells. Each result represents the mean ± SD of three experiments. Significance was calculated as *p value < 0.05 compared to vehicle control (fibrate-untreated uninfected U937 cells), using Student’s unpaired *t*-test. Significance was calculated as #*p* value < 0.05 compared to vehicle control (fibrate-untreated infected U937 cells) using Student’s unpaired *t*-test. Beza, bezafibrate; Feno, fenofibrate; Gem, gemfibrozil.

MyD88 increased in infected cells (Figure 4, panels a and b, lane 2) compared with that in the non-infected vehicle control group, whereas the protein level was not different in fenofibrate and gemfibrozil treated cells compared to that in the non-infected vehicle control at 1 and 6 h, respectively. MyD88 expression in the bezafibrate treated group was not significantly different compared to that in the vehicle control group at 1 h; however, it decreased to the non-infected vehicle control level at 6 h (1 h infection: infected vehicle control; 2.17 ± 0.20, *p* < 0.01, 24 h bezafibrate treatment; 2.00 ± 0.04, *p* < 0.01, 6 hr infection: infected vehicle control; 2.52 ± 0.19, *p* < 0.01) (Figure 4).

## Discussion

Tuberculosis is an infectious disease caused by *M. tuberculosis* that grows and divides in pleural macrophages. Fibrates are associated with the immune response and control of the intracellular lipid metabolism. In this study, we investigated the effect of fibrate pretreatment on the immune response during *M. smegmatis* infection in the U937 human leukemic monocyte lymphoma cell line. Our results suggest that fibrate pretreatment reduced the inflammatory stresses in *M. smegmatis*-infected U937 cells through a PPAR-α independent pathway.

ManA belongs to phosphomannose isomerase and is encoded by the *ManA* gene, which interconverts fructose-6-phosphate and mannose-6-phosphate [4]. Methionine amino peptidase is encoded by the *MetAP* gene and deletes the N-terminal methionine from polypeptides during protein synthesis in eukaryotes and prokaryotes [7]. Genes of both enzymes were found in *M. smegmatis* [4, 11]. In this study, *M. smegmatis* was collected from U937 cells exposed to *M. smegmatis* to confirm infection. The genomic DNA was then used in a PCR reaction with ManA and MetAP specific primers. Bands appeared at the same size as the control group; thus, confirming infection by *M. smegmatis* (Figure 1).

The effect of *M. smegmatis* infection on target protein expression in U937 cells was identified by Western blot. The expression of ACAT-1, TNF-α, PPAR-α, and MyD88 increased (Figure 2). Especially, the expression of MyD88, which binds to TLR2, a receptor of *Mycobacterium spp.* [29], markedly increased in *M. smegmatis*–infected U937 cells (Figure 2). Inflammatory responses to *M. smegmatis* infection could be predicted in U937 cells due to the elevated expression of TNF-α and MyD88.

Western blotting was performed using ACAT-1 antibodies on fibrate-treated U937 cells to examine the effect of bezafibrate, fenofibrate, and gemfibrozil on U937 cells (Figure 3). ACAT-1 participates in the degradation and formation of ketone bodies and cholesterol biosynthetic pathway [35, 36]. ACAT-1 protein expression was suppressed in fibrate pretreated U937 cells, which may represent increased lipid β-oxidation, and both cholesterol and ketone body synthesis were inhibited (Figure 3). However the ACAT-1 expression pattern did not change following *M. smegmatis* infection (Figure 4). Our results indicate that the *M. smegmatis* infection mechanism has no relationship with the cholesterol and ketone body synthetic pathway.

Fibrates are known to inhibit lipid synthesis and lipid metabolism via activation of PPAR-α [15, 37, 38] and effect of lipid metabolism during TB infection has been reported in numerous studies [16, 18, 19, 27]. Phagocytosis control, enabled by control of other lipid metabolism, can inhibit infection. The effects of fibrates, which are PPAR-α agonists, on PPAR-α expression in *M. smegmatis*-infected U937 cells was investigated. Their expression patterns were not so different under fibrate pretreatment; thus, the effect of PPAR-α signaling on the infection mechanism was unclear (Figure 4). Because it was reported and hypothesized that gemfibrozil blocked TB by inhibiting enoyl reductase [20] or by regulating cholesterol metabolism via RORγ inhibition in macrophages [39], other targets or mechanism of fibrates except for PPAR-α could be deduced.

The cells were pretreated with fibrates for 24 h prior to exposure to *M. smegmatis* for 1 or 6 hours to examine the effect of fibrate pretreatment on *M. smegmatis* infection in U937 cells. Western blotting was used to observe changes in the expression of inflammatory protein, MyD88 and TNF-α. Fibrate-treatment resulted in down-regulation of MyD88 and TNF-α expression at 1 and 6 h after infection in U937 cells (Figure 4). MyD88 is a messenger of TLR signaling during a TB infection [17, 29] and TNF-α is important in the macrophage infection mechanism via TB phagocytosis [40–42]. It was reported that fibrates could target membrane isoprenoid quinones and could show toxicity in *M. tuberculosis* [43], possibly resulting in the reduction of the burden of infection and inflammation. Since TNF-α and MyD88 have been regarded as a critical component of the inflammatory immune response to *M. tuberculosis*, the normalization of their expression possibly means the amelioration of pathologic condition of fibrate pretreated differentiated macrophages or the decrease in the infection rate.

## Conclusion

Our data suggest that fibrate pretreatment normalized the levels of inflammatory molecules in *Mycobacterium smegmatis*-infected U937 cells. The possible effect of fibrate treatment on *M. smegmatis* in macrophages was suggested here by examination of protein expression of ACAT-1, PPAR-α, TNF-α, and MyD88. New approaches using fibrates to evaluate TB infection could function to explain the mechanism of host immune response and the infection inhibition. Further studies are needed to establish the mechanism of action of fibrates in inhibiting *Mycobacterium spp.* infection.

## Methods

### Cell culture and differentiation

Human leukemic lymphoma U937 cells were cultured on 100 mm cell culture plates in RPMI 1640 medium (Caisson Labs, Logan, UT, USA) with 10% fetal bovine serum (Thermo Scientific, Waltham, MA, USA), 1% penicillin-streptomycin (Invitrogen, Carlsbad, CA, USA) and 1% sodium pyruvate (Welgene, Seoul, Korea) at 37°C in an incubator (Thermo Scientific) containing 95% air and 5% CO_2_. The cells were differentiated by treatment with phorbol myristate acetate (PMA, 10 μg/ml, Sigma, St Louis, MO) for 72 h [1].

### Bacteria

*Mycobacterium smegmatis* (mc^2^155), stored at −70°C, was thawed and then resuspended in RPMI 1640 medium for the infection test.

### M. smegmatis infection assayed by PCR

Differentiated U937 cells were exposed to *M. smegmatis* for 1 h (multiplicity of infection, MOI, 5:1 mycobacteria to cell ratio). The U937 cells were removed from the medium and washed with PBS three times, and dH_2_O was added to lyse the U937 cells through osmosis. The supernatant was transferred to new tubes and centrifuged. The resulting bacteria pellet was resuspended in Tris-EDTA buffer. The bacteria were boiled at 100°C for 3 min, and then PCR analysis was performed on their genomic DNA using the following sense and antisense primers: ManA, forward primer 5’-TCGACGGCGCGATCAACTAC-3’ (527–546), reverse primer 5’-ATCTCGTGTCCGAGCTGCTC-3’ (931–950); MetAP, forward primer 5’-CACGACCGCTGAACGAACTC-3’ (8–27), reverse primer 5’-GACGCTGGAGCAGTTCGTTG-3’ (640–659). The thermocycling protocol used for amplification of ManA and MetAP included 35 cycles of 95°C for 60 s, 58.5°C for 60 s, and 72°C for 1.5 min, followed by 10 min of final extension at 72°C. The PCR products were separated using electrophoresis on a 1.2% agarose gel and visualized by ethidium bromide staining.

### Western blotting

Differentiated U937 cells (2.5 × 10^6^) were cultured on 100 mm plates in medium and then mixed with either dimethyl sulfoxide (0.001% DMSO), as the vehicle control, or with 1 μM of each fibrate (bezafibrate, fenofibrate, and gemfibrozil) for 24 h at 37°C in an incubator (Thermo Scientific) containing 95% air and 5% CO_2_. After 24 h, the treated cells were exposed to *M. smegmatis* for 1 h or 6 h (MOI, 5:1). After washing with PBS, the proteins were extracted using RIPA buffer (Novagen, Madison, WI, USA). A protease-inhibitor cocktail (Sigma, St Louis, MO, USA Catalog Number P2714) was added at each step to extract the proteins. From each sample, 15–40 μg of protein was extracted and electrophoresed on a sodium dodecyl sulfate polyacrylamide gel (Bio-Rad, Hercules, CA, USA). The proteins were then transferred to a polyvinylidene fluoride membrane (Bio-Rad) for Western blot analysis. The protein-containing membrane was blocked using 5% skim milk (Bio-Rad) and then incubated with primary anti-ACAT-1 (Santa Cruz Biotechnology, Santa Cruz, CA, USA), anti-PPARα (Santa Cruz Biotechnology), anti-TNF-α (Santa Cruz Biotechnology), anti-MyD88 (Santa Cruz Biotechnology) or anti- β-actin (Sigma) antibodies. Subsequently, anti-mouse (Santa Cruz Biotechnology), anti-rabbit (Santa Cruz Biotechnology) or anti-goat (Santa Cruz Biotechnology) secondary antibodies was incubated with the membrane, and the protein band was visualized using Super-Signal West Pico Luminal/Enhancer solution (Thermo Scientific). All images were enhanced using Photoshop (Adobe Systems, San Jose, CA, USA).

### Statistical analysis

Experiments were performed in triplicate. Statistical significance was evaluated by Student’s t-test using the TINA 2.0 densitometric analytical system (Raytest, Straubenhardt, Germany). P values < 0.05 were considered significant.

## Authors’ information

Sung-Jo Kim and Minho Hong are co-first authors.

## Abbreviations

ACAT-1: Acetyl transferase-1
BCG: *Bacillus calmette-guerin*
DMSO: Dimethyl sulfoxide
ManA: Mannose-6-phosphate isomerase class I
MetAP: Methionine amino peptidase
MyD88: Myeloid differentiation primary response gene 88
PCR: Polymerase chain reaction
PMA: Phorbol myristate acetate
PPAR-α: Peroxisome proliferator-activated receptor-α
RXR: Retinoic acid receptor
TB: Tuberculosis
TLR: Toll like receptor
TNF-α: Tumor necrosis factor α.
